# Cartilage structure increases swimming efficiency of underwater robots

**DOI:** 10.1038/s41598-021-90926-9

**Published:** 2021-05-28

**Authors:** Masaki Yurugi, Makoto Shimanokami, Toshiaki Nagai, Jun Shintake, Yusuke Ikemoto

**Affiliations:** 1 Faculty of Science and Technology, Department of Mechanical Engineering, Meijo University, 1-501 Shiogamaguchi, Tempaku-ku, Nagoya, 468-8502 Japan; 2 Department of Mechanical and Intelligent Systems Engineering, The University of Electro-Communications, 1-5-1 Chofugaoka, Chofu, Tokyo 182-8585 Japan

**Keywords:** Soft materials, Mechanical engineering

## Abstract

Underwater robots are useful for exploring valuable resources and marine life. Traditional underwater robots use screw propellers, which may be harmful to marine life. In contrast, robots that incorporate the swimming principles, morphologies, and softness of aquatic animals are expected to be more adaptable to the surrounding environment. Rajiform is one of the swimming forms observed in nature, which swims by generating the traveling waves on flat large pectoral fins. From an anatomical point of view, Rajiform fins consist of cartilage structures encapsulated in soft tissue, thereby realizing anisotropic stiffness. We hypothesized that such anisotropy is responsible for the generation of traveling waves that enable a highly efficient swimming. We validate our hypothesis through the development of a stingray robot made of silicone-based cartilages and soft tissue. For comparison, we fabricate a robot without cartilages, as well as the one combining soft tissue and cartilage materials. The fabricated robots are tested to clarify their stiffness and swimming performance. The results show that inclusion of cartilage structure in the robot fins increases the swimming efficiency. It is suggested that arrangement and distribution of soft and hard areas inside the body structure is a key factor to realize high-performance soft underwater robots.

## Introduction

The ocean contains valuable resources such as mineral resources and marine life^1,2^. To explore them, underwater robots are useful as many areas in the ocean are inaccessible to humans. Traditional underwater robots use screw propellers^3–5^, which may be harmful to marine life due to noise and accidental entrapment^6,7^. In contrast, robots that incorporate the swimming principles, morphologies, and softness of aquatic animals are expected to be more adaptable to the surrounding environment. Hence, various types of soft biomimetic underwater robots have been developed. Among them, there are robots that mimic the propulsion mechanisms of aquatic animals^8–38^. Aquatic animals, specifically fishes have a wide variety of swimming forms. For example, swimming of Batoidea, a kind of stingrays is based on Rajiform^39–41^. This type of swimming is based on generation of traveling waves on flat, large pectoral fins. The plane morphology of Rajiform swimmer is expected to be suitable for moving around the seafloor, which would enable efficient exploration of mineral resources and marine life.

**Figure 1:**
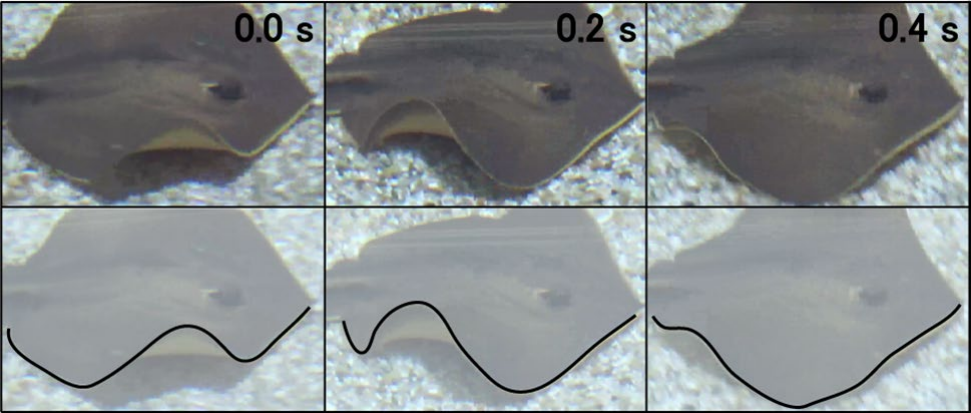
Swimming of a stingray (pictures are taken by the authors). Traveling waves are passing from anterior to posterior along the pectoral fins.

**Figure 2:**
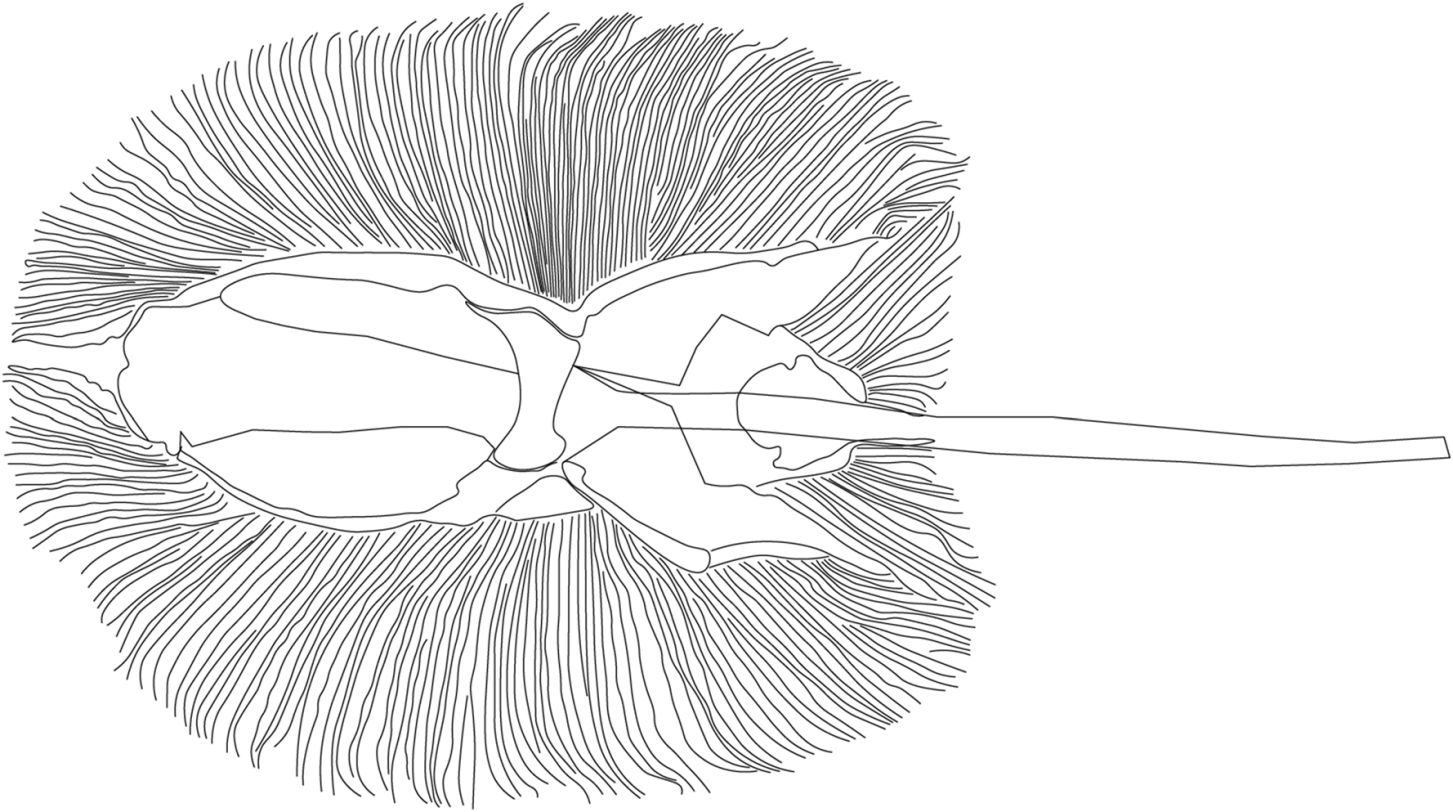
Skeleton diagram of stingray (drawn with reference to^52^). Cartilage structure is radially distributed in the body providing anisotropic body stiffness.

Stingray is the one of the Rajiform swimmers whose swimming behavior is shown in Fig. 1 in the form of sequential photos. The structural waves of the fins travel from the front to the back, generating thrust force in the forward direction. This suggests that the fins of stingrays are compliant in the primary swimming direction and relatively rigid in the horizontal perpendicular direction in order to transmit the momentum of traveling waves. From an anatomical point of view, the skeleton of stingrays is consisted of cartilages, an elastic tissue. As can be seen in Fig. 2, cartilage structures are radially distributed across the fins, resulting in anisotropic nature of their stiffness. We hypothesized that such anisotropy is responsible for the generation of traveling waves that enable a highly efficient swimming.

In this study, we validate our hypothesis through development of stingray robots with embedded cartilage structure. To the best of our knowledge, no study on the incorporation of cartilages has been reported, even though numerous stingray-like robots have been developed^42–51^. Our robots consist of silicone elastomers with the different bulk stiffness that represent soft tissue and cartilages. Hence, we investigate first time the effect of cartilage structure inclusion on the anisotropic stiffness in a soft structure by performing a tensile test. Next, we fabricate stingray robots using the materials characterized in the tensile test. We conduct customized bending test of the robots to confirm the overall stiffness of the body. Then, we show experimentally that stiffness anisotropy of the fins realized by the cartilage structure can increase the swimming efficiency even though the overall stiffness of the robots remains the same.

## Results

### Bulk bending test

Specimens were fabricated and the tensile test was performed to investigate the effect of cartilage structure inclusion on anisotropic stiffness. Specimens of two types of silicone elastomers were tested: Ecoflex 00-20 (Smooth-On) and Sylgard 184 (Dow Corning). The former was a compliant elastomer (ultimate tensile strength ~ 1.1 MPa) used as the soft tissue. The latter was a rigid elastomer (ultimate tensile strength ~ 6.7 MPa) used as the cartilage.

**Figure 3:**
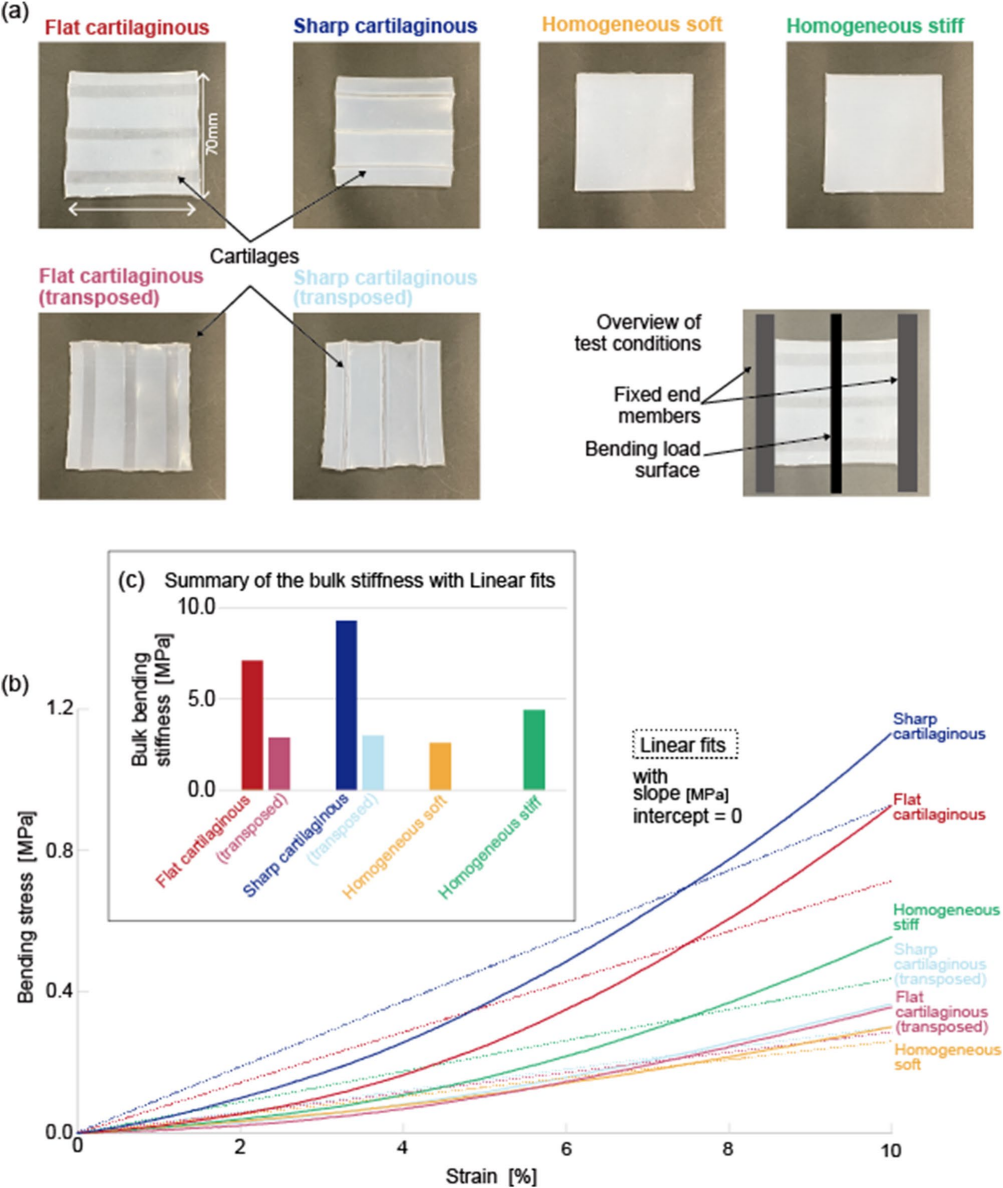
Results of bulk bending tests of specimens. (**a**) Top view of six types of specimens: Flat cartilaginous, Sharp cartilaginous, Homogeneous soft, Homogeneous rigid, and Flat cartilaginous, Sharp cartilaginous consisting of the same silicone elastomers taken for comparison. The composition and structure of Flat cartilaginous (Sharp cartilaginous) and Flat cartilaginous (Sharp cartilaginous) samples are the same, whereas the direction of cartilages is parallel and perpendicular to the strain direction. Sharp cartilaginous with a height of 6 mm consist of the cartilages with a sharper shape as compared to Flat cartilaginous with a height of 1 mm. Homogeneous soft sample is without cartilages, whereas Homogeneous rigid is a mixture of the soft tissue and cartilage materials. (**b**) Stress–strain curves of the specimens. The solid lines are the measured data, and the corresponding dashed lines are linear approximations. (**c**) The bulk stiffness of each specimen obtained as the stress to strain ratio at 1% stress.

Based on these materials, four types of specimens were prepared as shown in Fig. 3a: “Flat cartilaginous” specimen with soft tissue and cartilages, “Sharp cartilaginous” specimen with soft tissue and cartilages which has relatively narrow width and large thickness, “Homogeneous soft” specimen consisting of soft tissue only, and “Homogeneous rigid” specimen consisting of randomly arranged cartilage materials in the soft tissue. In the specimen Homogeneous rigid, the amount and mass ratio of the materials are the same as those in the Flat cartilaginous and Sharp cartilaginous. Every specimen had the dimensions of 70 mm (width) × 70 mm (length) × 3 mm (thickness). The results of the bulk bending test are shown in Fig. 3b. The details of this experiment are explained in the Method section. The bulk stiffness values of each specimen obtained from the experimental data are expressed as the slope of a linear approximation curve, and summarized in Fig. 3c. The data showed that Sharp cartilaginous specimen has the largest stiffness among the samples (9.3 MPa), followed by Flat cartilaginous (7.1 MPa). Homogeneous soft and Homogeneous stiff exhibited lower stiffness: 4.4 MPa and 2.6 MPa, respectively. The bulk bending stiffness of Flat cartilaginous and Sharp cartilaginous in the transposed direction are 2.8 MPa and 3.0 MPa, respectively. These values are the close to the value of Homogeneous soft (2.6 MPa). The difference in the bulk stiffness modulus in the specimens was clearly correlated with the presence of the cartilage structure. This result also suggests that the anisotropy of stiffness is caused by the alignment of cartilages.

### Fabrication of the robots

**Figure 4:**
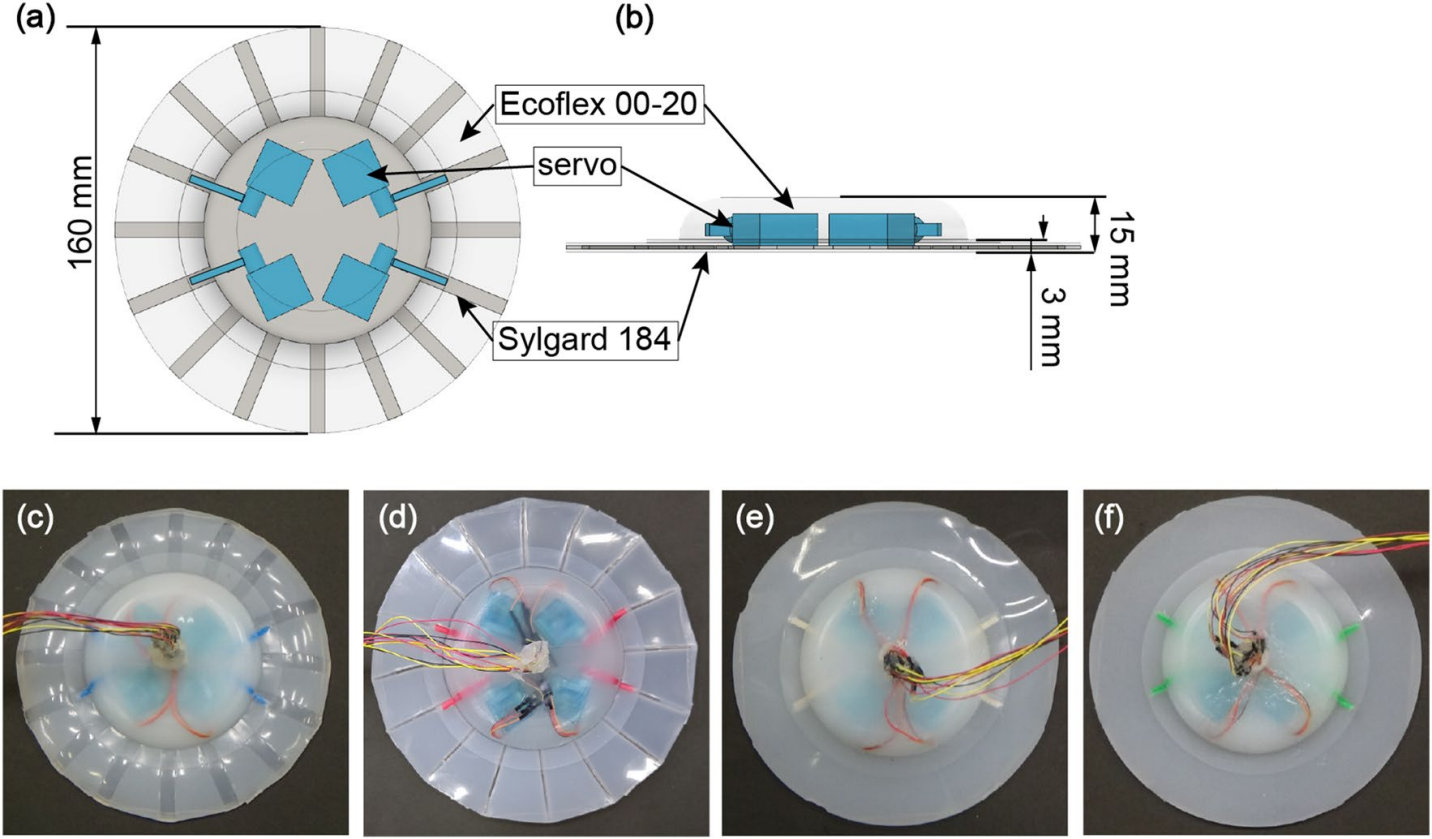
Overview of a developed robot consisting of a soft tissue (Ecoflex 00-20, Smooth-On), servomotors (FS 0403, FEETECH), and cartilages (Sylgard 184, Dow Corning): (**a**) top view of the robot structure; (**b**) side view of the robot structure; (**c**) the robot with flat cartilaginous; (**d**) robot with sharp cartilaginous; (**e**) robot made of homogeneous soft material; (**e**) robot with homogeneous stiffness, made of the mixture of cartilage and soft tissue materials.

Figure 4a,b illustrates the structure of the robot having a circular shape with a diameter of 160 mm and a fin thickness of around 3 mm. The robots were made of the three main parts: the cartilages, the body (soft tissue), and the servomotors (FS0403, FEETECH). The servomotors were powered externally through electrical wires. The servomotors were coated with a silicone bond (BathbondQ, KONISHI) to ensure water resistance. The use of waterproof servomotors can also be considered. However, they were not used in this study because of the robot size limitation. A pressure-tube consists of rubber, and very light and bond coated plug joint was used to prevent short circuits. We developed four types of stingray robots based on the same set of materials used in the bulk bending tests explained in previous section. The first was with the Flat cartilaginous (Fig. 4c), in which the cartilages were 1 mm thick and 6 mm wide. The second was with the Sharp cartilaginous (Fig. 4d), in which the cartilages were 6 mm thick and 1 mm wide. These robots consist of materials with the same weight ratio, and allow comparing the effects of different cartilage shapes on swimming efficiency. The third one, Homogeneous soft (Fig. 4e), was without cartilages structures. In the last one, the amount and ratio of the materials were the same as in the Flat and Sharp cartilaginous corresponding to Homogeneous stiff (Fig. 4f). The cartilages were designed by the analogy with the actual skeleton of a stingray that has radially distributed cartilage structure.

**Figure 5:**
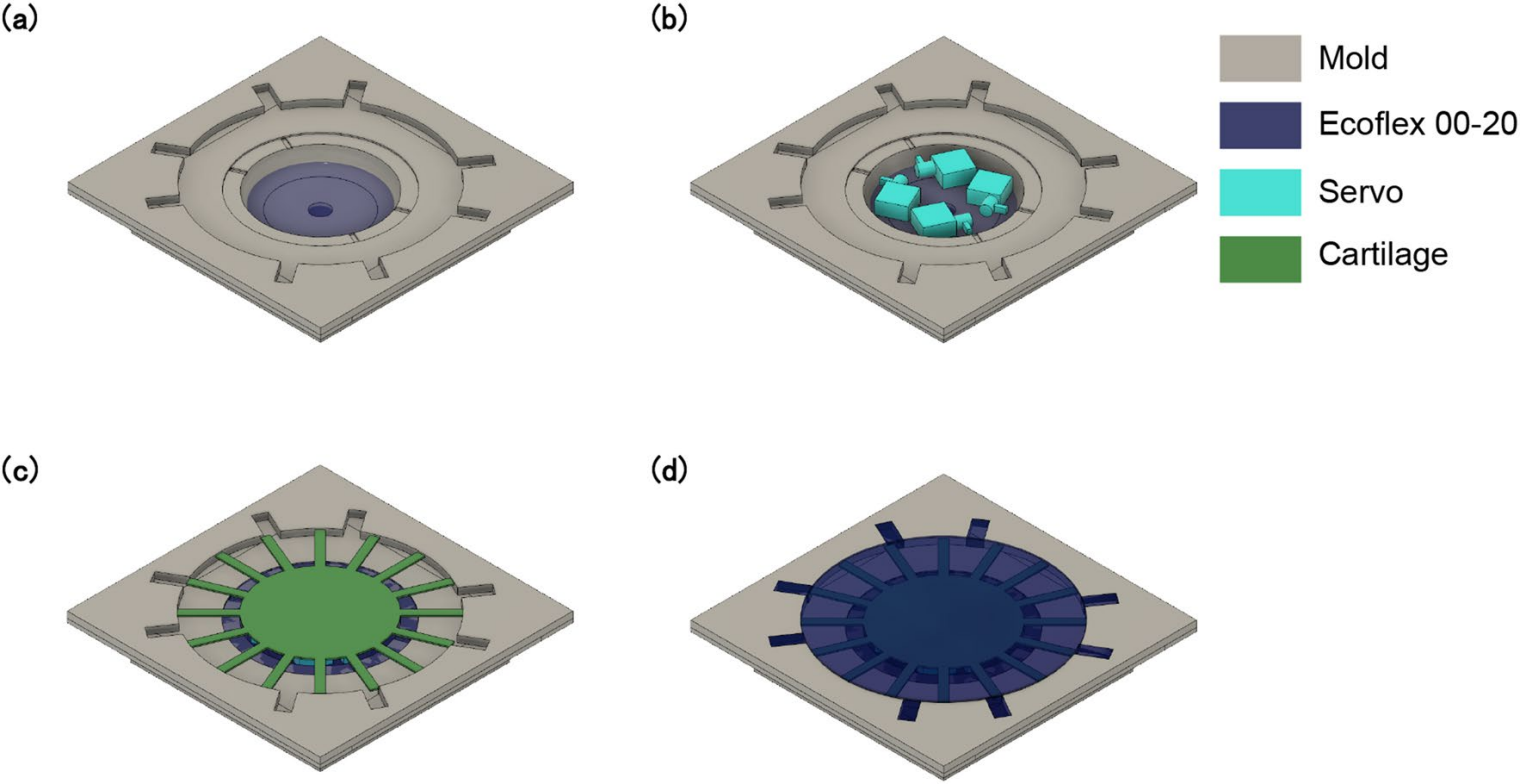
Robot fabrication process based on molding. The mold is made of multiple acrylic parts: (**a**) Ecoflex 00-20 is casted on a mold and cured in an oven; (**b**) servomotors are placed inside the mold; (**c**) cartilages made of Sylgard 184 are placed above the servomotors (cartilages are fabricated separately in another mold); (**d**) mold is filled with Ecoflex 00-20.

For the fabrication of robot body and cartilages, the molds were used to solidify the liquid materials. The molds were made of an acrylic plate, and consisted of multiple parts. A CNC router (MDX-540S-AP, Roland) and a laser cutting machine (Speedy 360, Trotec) were used to produce the molds. Figure 5 summarizes the fabrication process of the robot based on the molding. The cartilage parts were fabricated by casting Sylgard 184 into the mold, and then solidification in an oven at 80 °C for 1 h. Bubbles inside the silicone were removed using a vacuum chamber. For fabrication of the robot body, Ecoflex 00-20 was poured into the mold to fix the servomotors, cartilages, and other parts. After the silicone was completely solidified, the robot was removed from the mold.

### Customized bulk bending test

**Figure 6:**
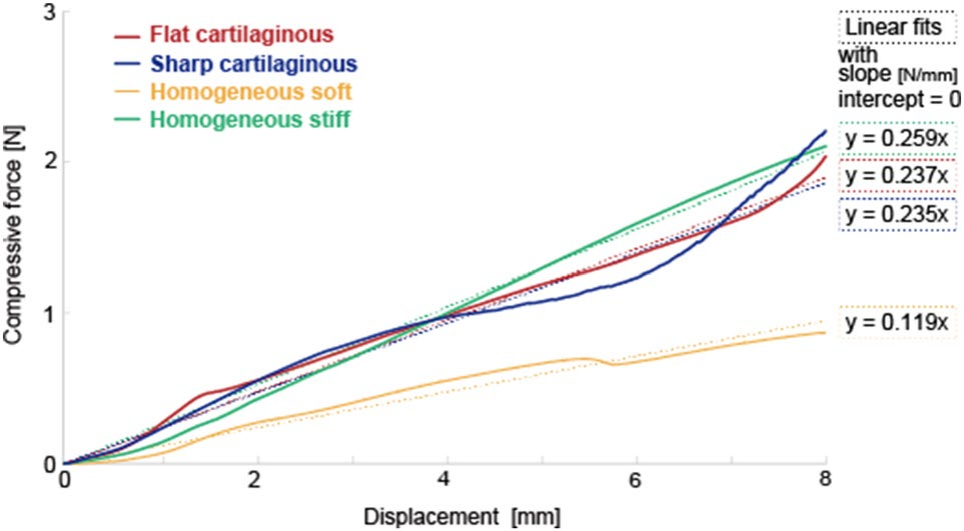
Result of customized bulk bending test of the fabricated robots. Solid lines represent the measured data, and dashed lines are their linear approximations.

We performed customized bulk bending tests of the robots to examine their overall stiffness (see the Method section for the details of the experiment). Figure 6 shows that the stiffness of the robot with Flat cartilaginous is similar to that in both Sharp cartilaginous and the robot with Homogeneous rigid made of the mixture of soft tissue and cartilage materials. In contrast, the robot with Homogeneous soft made only of soft tissue displays much lower stiffness. Therefore, by comparing the performance of these four robots it is possible to distinguish the effect of cartilage inclusion on the swimming efficiency disregarding the overall stiffness. According to the results of bulk bending test as shown in Fig. 3 and customized bulk bending test, the same trend was observed: the stiffness in the circumferential direction tended to be the same for the materials with the same weight mixing ratio.

### Swimming test

**Figure 7:**
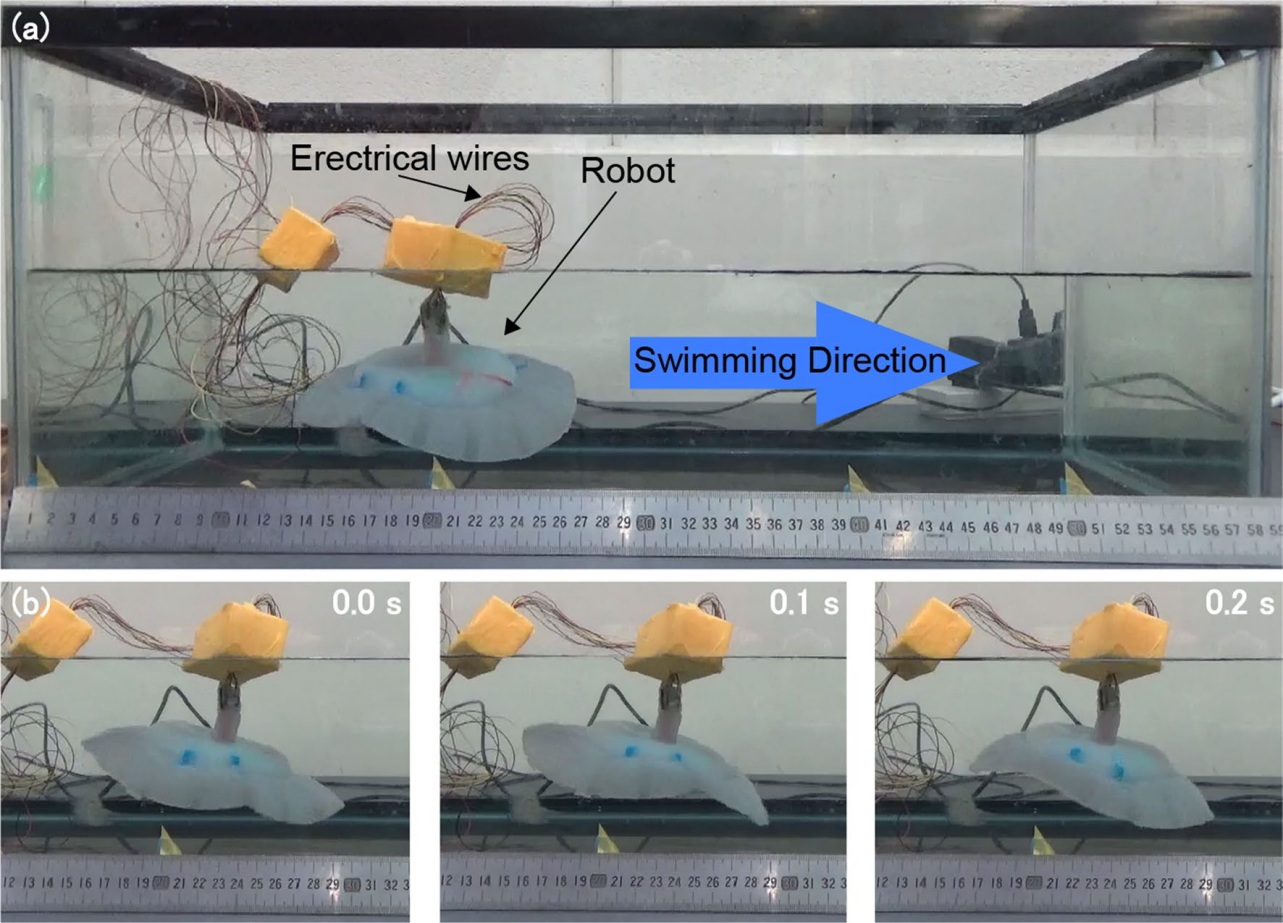
(**a**) Experimental setup to measure the swimming speed and power consumption of the robots; (**b**) sequence of swimming movements of a robot with cartilage structure. Traveling waves are generated by oscillating servomotors from the front to the back of the pectoral fin, thereby realizing swimming of the robot.

We conducted swimming test of the developed robots in the experimental environment shown in Fig. 7a where the robot is immersed in a water tank filled with tap water. Figure 7b depicts a sequence of swimming movements of a robot with cartilage structure. Following the expectations, traveling waves were generated along the fins that push the robot forward. We measured the swimming speed and electric power consumption for each type of the robots while varying the frequency of the 7.5 V driving voltage from 0 to 9 Hz with 1 Hz increments. At every tested frequency, the measurements were performed 10 times and the average value was reported.

**Figure 8:**
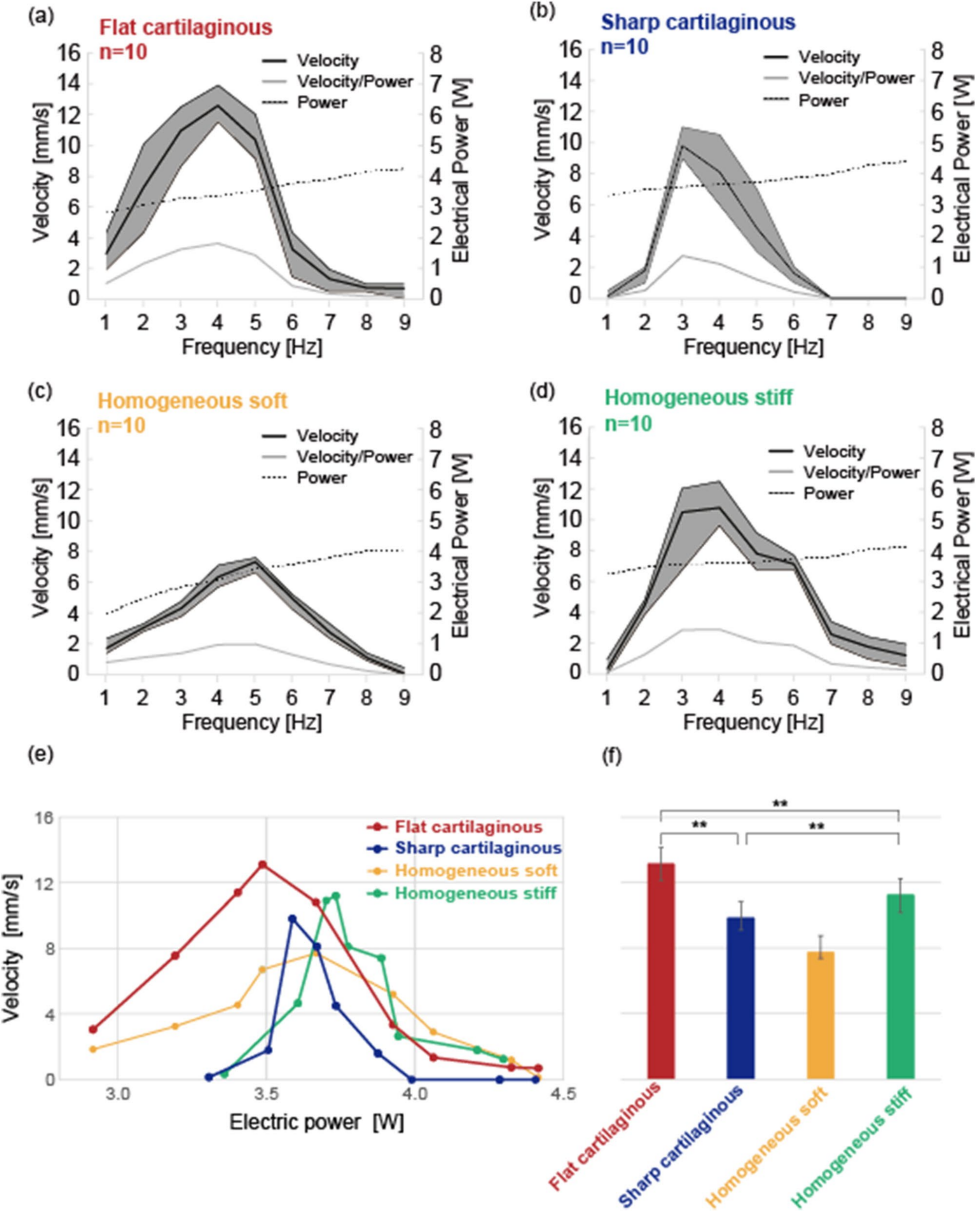
Measured swimming speed and electric power consumption as functions of the driving frequency for: (**a**) robot with flat cartilaginous, (**b**) robot with sharp cartilaginous, (**c**) robot made of homogeneous soft material, and (**d**) robot with homogeneous stiffness made of a mixture of soft tissue and cartilage materials. (**e**) Swimming speed as a function of the electric power. (**f**) Summary of the peak velocities at the optimal frequencies following Figs. (**a**–**d**). Significant differences in the average velocity according to ANOVA test are (p < 7.5e10^−8^) between the flat and sharp cartilaginous, (p < 4.9e10^−4^) between the flat cartilaginous and the homogeneous stiff cartilaginous, and (p < 2.5e10^−3^) between the sharp and the homogeneous stiff cartilaginous. The robot with flat cartilage structure exhibits the highest speed and the lowest power consumption indicating the effect cartilage structure inclusion on the swimming efficiency.

On the whole, the swimming speed increased compared to the robot with Homogenous soft as shown in Fig. 8a–d and summarized in Table 1. The most efficient swimming was realized by the robot with Flat cartilaginous. Then, a peak speed of 13.1 mm/s appeared at a frequency of 4 Hz. The peak electric power consumption was 3.48 W. The presence of peak in the swimming speed suggests that there is a resonant frequency of the structure which enhances the amplitude of traveling waves. Regarding the robot without cartilage structure, Homogeneous soft, the swimming speed was lower compared with the other robots as can be seen in Fig. 8c,d. Because the fin was soft, it may be difficult to spread driving force from the servos over the entire fin. Therefore, it is expected that too soft fins would not generate a traveling wave well enough for efficient swimming. Interestingly, the speed of the robot with Homogeneous rigid is comparable to the one with Sharp cartilaginous. This suggests that a certain body stiffness is important in fish-like swimming, in addition to the presence of the cartilage structure. When comparing the robots in terms of swimming speed as a function of the power consumption, the effect of cartilage structure is more visible. As shown in Figure 8e, the robot with Flat cartilaginous is much more efficient than the robot with Sharp cartilaginous, Homogeneous stiff, and Homogeneous soft. Interestingly, the efficiency of Sharp cartilaginous and Homogeneous stiff are more or less the same as summarized in Figure 8f. This is analogy with the results obtained for the swimming speed. These results suggest that the robot with cartilage structure can improve swimming efficiency, and there could be optimal size and shape of cartilaginous.

**Table 1:** Summary of peak speed, electric power, and the corresponding driving frequency for the swimming tests.

	Flat cartilaginous	Sharp cartilaginous	Homogeneous soft (only soft tissue)	Homogeneous stiff (mixture of cartilage and soft tissue materials)
Maximum Speed [mm/s] (SD)	13.1 (0.92)	9.8 (0.68)	7.7 (0.33)	11.2 (0.98)
Electrical Power [W] (SD)	3.48 (3.23)	3.58 (3.3)	3.64 (3.34)	3.73 (3.38)
Frequency at which maximum speed is taken [Hz]	4	3	5	4

## Discussion

Following the similar overall stiffness of the robots with cartilage structure and that made of the mixture, the result validates our hypothesis that the cartilage structure enhances the swimming efficiency of underwater robots based on Rajiform swimming. The peak speed and electric power for the tested robots are summarized in Table 1. The result also suggests that anisotropic arrangement of soft and hard domains, as represented by soft tissue and cartilages in the robot, are important structural parameter that may define the swimming behavior, as well as enhance swimming efficiency. In the case of Rajiform swimming, the body stiffness direction can affect the swimming performance. The developed robot consists of radially aligned cartilage structure, where the body stiffness is soft in the circumferential direction and hard in the radial direction. The circumferential softness is necessary to generate traveling waves across the body. The radial hardness can be advantageous to transmit mechanical power across the body. Thereby, our results can be summarized as follows: the cartilage structure enables localization of the body stiffness. Furthermore, appropriate design of the cartilaginous shape including stiffness gradient enables a highly efficient swimming.

Compared to the subcomponents, the cartilage structure density in the robot in this study decreases in the radial direction causing softening from the center outwards. This fact highlights the importance of the stiffness gradient effects on the motion. Despite the results in this study are obtained with a specific robot, a possibility of swimming rate increase due to cartilages structure is shown.

In general, for a soft robot, the softness is a significant advantage to achieve a skillful motion. Simultaneously, too high softness does not allow to transmit driving force through the body. One way to improve the body stiffness is to use materials made with a mixture of hard materials. However, the design of a body structure would be limited because the body consists of homogeneous material and the stiffness is ubiquitous throughout the body. Our results represent another way for improving the stiffness of a soft robot, where a properly arranged hard material incorporated into a soft one. In this study, the proposed local arrangement of cartilage structure worked well for swimming. It is expected that the cartilaginous is relatively stiff as well as elastically deformable, so its stiffness can be locally adjustable. In other words, when the properties of the material itself are limited, the structure arrangement of multiple materials may be the solutions to fabricate a soft robot with variable local softness, which generates efficient motion.

Future work will focus on investigation of the effect of anisotropic stiffness in different soft robotic platforms, and establishing the ways to design such robots with optimized geometry and modulus of materials to control the stiffness. Nevertheless, we believe that demonstrated arrangement and distribution of soft and hard domains in the structure can be a promising approach to designing high-performance underwater soft robots.

## Methods

Table 2 shows the properties of the materials used. Both of the silicone elastomers employed are two-component liquid mixtures. The material was mixed by hand to prevent mixture separation. Specific weights of both materials are slightly greater than that in water. Sylgard 184 is harder than Ecoflex 00-20 because of its higher tensile strength and hardness (Shore A) values. The silicone elastomers were prepared as the follows. Ecoflex 00-20 was a two-component liquid mixture fabricated in a 1:1 weight ratio as recommended by the producer. Sylgard 184 was mixed with the main agent and the curing agent by the same procedure at a weight ratio of 10:1. Sylgard 184 possesses a low viscosity and has a tendency to penetrate into small crevices. During the mixing, air bubbles were incorporated into the mixture. Those bubbles were removed by placing the mixture in a vacuum vessel at a negative gauge pressure of 0.1 MPa. The mixture was then taken out of the container and poured into the mold. The soft material was cured on a horizontal surface. A thin layer of mold lubricant (Shin-Etsu Silicone, KS702-1) was applied to the mold to facilitate removal of the soft material after the curing. The curing can be accelerated by heating; however, in this study the robots were cured at room temperature (around 25 ℃) to avoid deformation owing to residual stress.

**Table 2:** Details of body tissue and cartilage.

	Body tissue	Cartilage
Material	Ecoflex 00-20	Sylgard 184
Ultimate tensile strength	1.1 MPa	6.7 MPa
Stiffness (Shore A)	0–20	43
Specific weight	1.4	1.03

**Figure 9:**
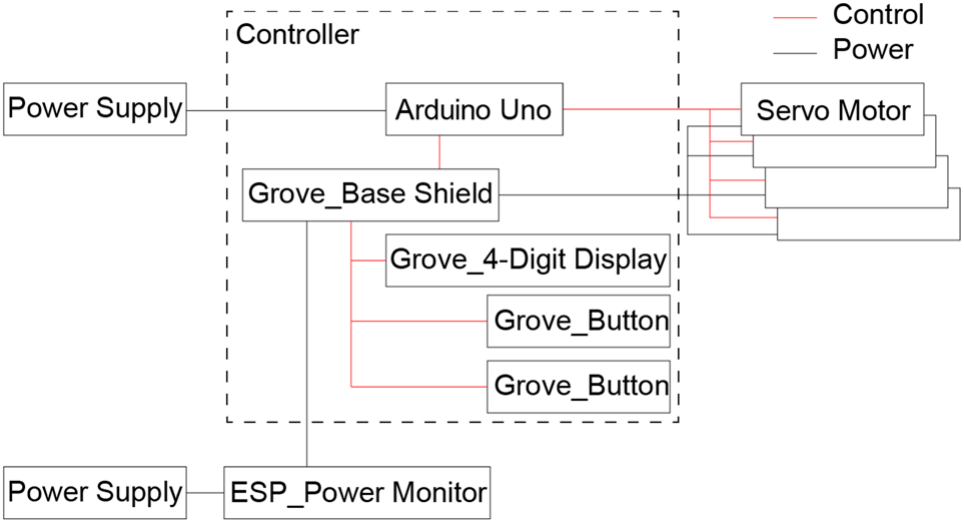
Power monitor and servomotor control diagram. The black line is the power supply electrical wires. The red line is connection for controls the electrical wires. The dashed line depicts the controller components.

Figure 9 represents a diagram of the system configuration used in this study. The servomotors are controlled by an Arduino Uno microcontroller. A microcontroller was supplied with 7.5 V, 0.8 A electric power. The electric power supply to the Arduino, an ESP_Power Monitor, and an electric power measurement device, was provided separately. Electric power supplied to the servos can be measured more accurately by using the ESP_Power Monitor only. A Grove_4-Digit Display and a Grove_Button were included into the system to check and change the frequency of the servomotors. A Grove Base Shield can be installed on the Arduino. However, because the separate electric power supply was needed as mentioned earlier, the shared pins of electric supply were removed. With this system, output parameters such as movement angle and frequency can be adjusted individually for each of the four servomotors. The buttons allow the user to change the servomotor frequencies. Frequency of the servos are displayed on the monitor. In the microcomputer program, the power supplied to the robots during 10 s. In each test, at the initial state, the robot was stopped, and the time of the servo switching on was set as zero, and the distance travelled was measured. The speed was calculated from the travelled distance for the specified time. This method of test took in to account the inertial drift after switching off the servos.

The input voltage phase shift was π/4 between the front and rear servos, so that the fins could generate a traveling wave. The peaks may shift if to apply another phase shift. However, in this study, the phase shift was fixed in all the experiments for accurate comparison of the effect of body stiffness on swimming efficiency. In the preliminary experiments, it was confirmed that the robot can move forward when the phase difference was below π/2 rad, so the π/4 rad shift was chosen within the range between 0 and π/2 rad. Power consumption was measured using the Power Monitor based on an INA 219 board and recorded at a period of 1 ms.

**Figure 10:**
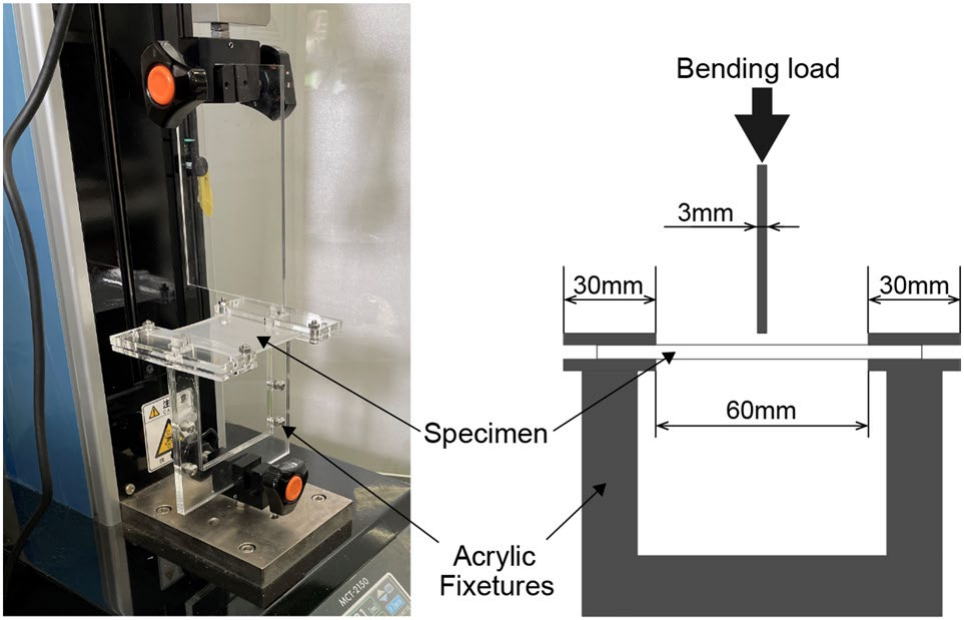
Overview of bulk bending test using a (MCT-2150) equipment.

To investigate the more basic properties of the material, specimens with cartilages structure similar to that of a robot were fabricated in advance. In this study, the bulk bending tests were conducted to investigate the stiffness of the robots. Figure 10 shows a test scene of the bulk bending test. In bulk bending test, so we fabricated fixture with acrylic plates because the specimens could not be directly fixed to measuring instrument. The test pieces had the dimensions of 70 × 70 × 3 mm3, where the part of bending occupies 60 mm inside the specimens since both sides of the specimen are sandwiched between the acrylic fixtures. The bending surface area was accurately standardized for all experiments to prevent the effect of deflection caused by its own weight as much as possible.

**Figure 11:**
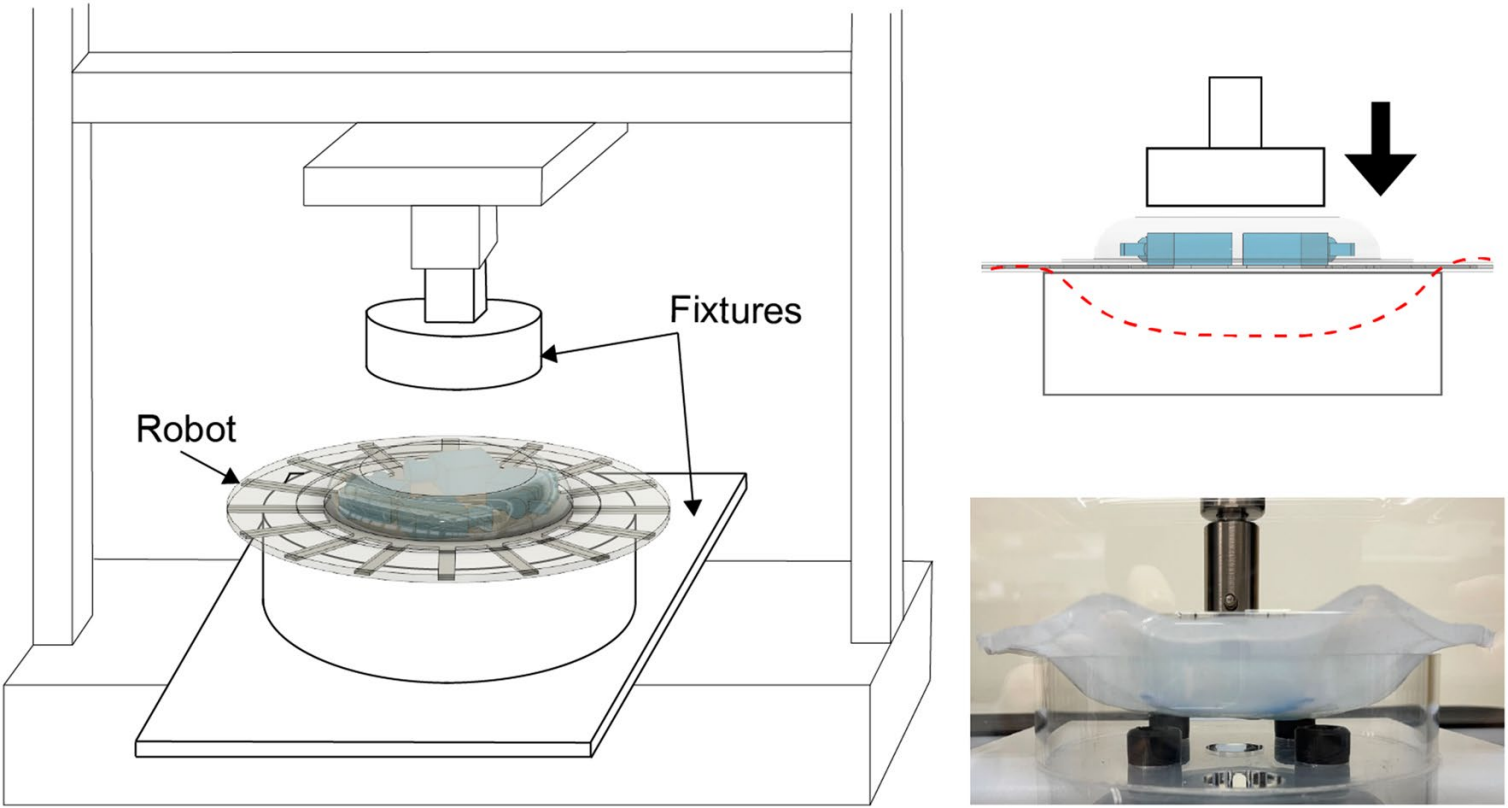
Customized bending test for robots performed with an AGS-20NX.

The fixtures were fabricated to fix the robots in the compression test as shown in Fig. 11. Joints to the load cell were fabricated with a polylactide and the other parts were fabricated of acrylic. The test speed was 10 mm/min, and the test stroke was 10 mm away from the place of contact with the robot.
